# Learning-Based Repetitive Control of a Bowden-Cable-Actuated Exoskeleton with Frictional Hysteresis

**DOI:** 10.3390/mi13101674

**Published:** 2022-10-04

**Authors:** Yunde Shi, Mingqiu Guo, Chang Hui, Shilin Li, Xiaoqiang Ji, Yuan Yang, Xiang Luo, Dan Xia

**Affiliations:** 1 Department of Mechanical Engineering, Southeast University, Nanjing 210096, China; 2 Department of Mechanical Engineering, Tsinghua University, Beijing 100084, China; 3 Shenzhen Institute of Artificial Intelligence and Robotics for Society, The Chinese University of Hongkong, Shenzhen 518172, China; 4 Key Laboratory of Micro-Inertial Instrument and Advanced Navigation Technology, Ministry of Education, School of Instrument Science and Engineering, Southeast University, Nanjing 210096, China

**Keywords:** iterative learning control, Bowden cable transmission, frictional hysteresis, gearbox backlash, phase-lead compensation, soft exoskeleton robot

## Abstract

Bowden-cable-actuated soft exoskeleton robots are known for their light weight and flexibility of power transmission during rehabilitation training or movement assistance for humans. However, friction-induced nonlinearity of the Bowden transmission cable and gearbox backlash pose great challenges forprecise tracking control of the exoskeleton robot. In this paper, we proposed the design of a learning-based repetitive controller which could compensate for the non-linearcable friction and gearbox backlash in an iterative manner. Unlike most of the previous control schemes, the presented controller does not require apriori knowledge or intensive modeling of the friction and backlash inside the exoskeleton transmission system. Instead, it uses the iterative learning control (ILC)to adaptively update the reference trajectory so that the output hysteresis caused by friction and backlashis minimized. In particular, a digital phase-lead compensator was designed and integrated with the ILC to address the issue of backlash delay and improve the stability and tracking performance. Experimental results showed an average of seven iterations for the convergence of learning and a 91.1% reduction in the RMS tracking error (~1.37 deg) compared with the conventional PD control. The proposed controller design offers promising options for the realization of lightweight, wearable exoskeletons with high tracking accuracies.

## 1. Introduction

Exoskeleton robots are wearable supporting structures designed for human strength enhancement, movement assistance, or rehabilitation training [1]. The structures of early exoskeletons are usually rigid and bulky, which are installed parallel to the human limbs [2]. The well-known Berkeley lower extremity exoskeleton (BLEEX) and Cyberdyne hybrid assistive limb (HAL) are of this type [3,4]. There have been various studies on the dynamical modeling and control of these rigid exoskeletons [5]. However, the rigidity and bulkiness of these exoskeletons severely limit the natural movements and wearing comfort of human body, which could even cause injuries to the human limbs during operation [6].

In light of the drawbacks of traditional rigid exoskeleton designs, soft exoskeletons have been developed recently for enhanced user comfort and wearing safety [7]. Tsagarakis et al. used linear pneumatic muscle actuators (PMA) as power sources for aseven-degree-of-motion prototype upper-arm training/rehabilitation exoskeleton system. With the excellent power-to-weight ratio, this type of actuator offers safety, simplicity, and lightness to the overall design [8]. Zhang et al. proposed acurved pneumatic muscle-based rotary actuator for a wearable elbow exoskeleton. Compared with the general utilization of PMA in a rotary joint, this new structure weakens the coupling relationship between the output torque/force and contacting displacement of the PMA so that it can be easily deployed in telerobotics with torque/force-feedback or applied in rehabilitation [9]. The pneumatic artificial muscles, however, exhibit a complex, nonlinear relationship between air pressure and contraction length, which is difficult to model accurately [10]. In addition, the creep effect of pneumatic muscles caused by friction between the braided mesh and rubber tube during contraction and diastole impacts the output force adversely [11].

The Bowden cable actuation system offers an alternative solution to the light-weight and portable realization of soft exoskeleton robots [12,13]. Asbeck et al. proposed a wearable soft exosuit driven by Bowden cables, where the motion of geared motors was transmitted by the relative movement between the inner cable and outer sheath to individual exoskeleton joints [14]. Such a design allows the power source to be placed away from the exoskeleton joints and in a more flexible manner. So far, Bowden transmission cables have been applied to soft exoskeletons on various human parts [15,16], such as elbows, hands, ankles, knees, and hips. Soft exoskeletons, however, impose particular challenges for precise tracking because of the nonlinear stiffness in the human–exoskeleton interface and varying characteristics of the entire system [17,18].

The nonlinear friction in the Bowden inner cable and outer sheath is amajor source of hysteresis and phase delay of the transmission system. Studies have shown that a significant amount of the motor torque is generated to overcome the friction of the Bowden transmission cable [19].One way to address this problem is to use servo motors with very high power output and fast responses, which could reduce the influence of frictional disturbance of the Bowden cables to some extent, even when a basic PID controller is used. Such an approach, however, usually requires a large off-board platform to mount the bulkyservo motors and amplifiers, which prevents the outdoor and portable application of the soft exoskeleton system [20].

As a result, much research work has been conducted on the accurate modeling and proper compensation of the frictional effect [21,22] to ensure the precise operation of the Bowden-cable-actuated soft exoskeleton. Zhang at al. presented a tendon-sheath artificial muscle based on the Hill muscle model, anda compound tendon-sheath artificial muscle transmission system similar to the form of antagonist muscles was modeled based on the static Coulomb friction model [23]. However, the proposed transmission model was based on the hypothesis of invariant curved path and the uniform curvature of the tendon sheath, which may not fully match real situations when applied. Dinh et al. introduced an algorithm for backlash hysteresis compensation based on the normalized Bouc–Wen model [24,25] to drive a soft exosuit for assisting elbow motions [26]. A custom-designed test bench was used to validate the proposed control paradigm and RMSE (root mean square error) in trajectory tracking around 1 deg was reported in their study. However, many parameters of the proposed method depend on the curvature angle of the Bowden cable and high computational complexity was involved, which limits the real-world embedded application where complex and configuration-dependent backlash hysteresis arises. Jeong et al. proposed a method which enables controlling the transmission of a Bowden cable without directly measuring the output tension [27]. In their study, the bend angle was primarily used for friction compensation, which was estimated based on the Bowden-cable angle (BoA) sensor [28] and the input tension of the actuation wire. Feedforward control of the output tension of the Bowden-cable transmission was then implemented using the proposed friction compensation algorithm for different shapes of the cable. Experimental tests verified the proposed method on a fixed test bench. However, the addition of BoA sensors to the Bowden cables sheath could lead to increased complexity and lower reliability of the overall exoskeleton system. Wang et al. designed a disturbance observer (DOB) to compensate for frictional force disturbances and parameter perturbations in the inner loop of the human–exoskeleton system. In addition, admittance control integrated with the human–exoskeleton interaction feedforward model was used to overcome the limitation of the force loading caused by the friction of the Bowden cable [29]. This method, however, heavily depends on the accurate modeling of the human–exoskeleton system, where uncertain model errors could easily send the control system unstable.

Recently, there have been some studies on the learning control of exoskeleton robots. Wang et al. applied the ILC algorithm with a D-type updating law to the shank part of an exoskeleton. As the model non-linearity of the exoskeleton robot leads to degradation in the efficiency of normal iterative learning methods, a feedback linearization method was introduced to improve the overall performance of the algorithm [30]. The study, however, was mostly restricted to theoretical simulations where no experimental validation was provided. Chen et al. combined the higher-order ILC and active disturbance-rejection control (ADRC) for a wearable hand-rehabilitation robot [31]. However, detailed stability analysis of the higher-order ILC was not elaborated in their work. Meng et al. developed a robust iterative feedback tuning (IFT) control for a parallel ankle rehabilitation robot actuated by pneumatic muscles [32]. However, the performance of the IFT technique in their study was sensitive to the specific optimization algorithm being used Ajjanaromvat et al. developed an online iterative learning linear quadratic regulator (OILLQR) and tested the algorithm on their AIT leg rehabilitation exoskeleton [33]. However, the experimental platform in their study was a traditional rigid exoskeleton, which does not share the particular issues of soft exoskeletons. Chen et al. proposed aparameter-optimal iterative learning-control (POILC) method, which was able to adjust the learning rate for each iteration [34]. The reported RMS tracking error, however, was relatively high (>6 deg), which still leaves room for further improvement.

This paper proposed the design of a learning-based repetitive controller, which could compensate for the nonlinear cable friction and gearbox backlash in an iterative manner. Unlike traditional control approaches, the presented controller does not depend on the complex modeling or a priori knowledge of the friction and backlash inside the exoskeleton transmission system. Conversely, an iterative learning control (ILC) scheme was proposed to adaptively update the command trajectory which minimizes the output hysteresis caused by cable friction and gearbox backlash. Particularly, a digital phase-lead compensator was designed and integrated with the ILC to solve the problem of backlash delay. With the proposed phase-lead iterative learning control (PLILC) design, the tracking performance of the knee angle was greatly improved compared with conventional PD control, eliminating the need of precise modeling or sophisticated compensation.

The rest of the paper is organized as follows. Section 2 describes the soft knee exoskeleton design. Section 3 is about the mathematical modeling of exoskeleton knee joint, with the Bowden cable transmission system. The learning-based repetitive controller design is presented in Section 4. The experimental results are provided in Section 5 with the analysis of tracking error and discussion on the phase-lead compensator, followed by our conclusions in Section 6.

## 2. Soft Knee Exoskeleton and Actuator Design

### 2.1. Soft Knee Exoskeleton

**Figure 1:**
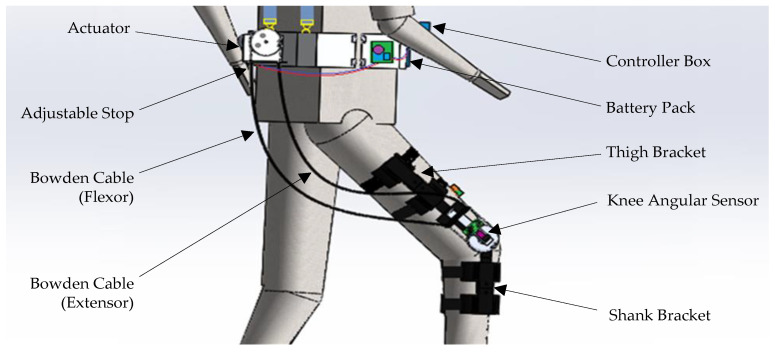
Overall design of the soft knee exoskeleton with the Bowden cable transmission system worn by the user.

The proposed soft-knee exoskeleton is illustrated in Figure 1 below. Structurally, the exoskeleton consists of the actuation stage, the Bowden transmission cables, the thigh and shank brackets, the knee angular sensor, the battery pack, and the controller box. The Bowden cables transmit the rotational displacement of the actuator to the exoskeleton knee joint through the relative movement of an inner cable inside the outer sheath in a flexible manner. As a result, the actuators, controller box, battery pack, and other components could be mounted away from the knee joint and near the user’s center of gravity. Additionally, the power of the electric actuator could be transmitted to the exoskeleton knee joint mechanism through the Bowden cable.

As shown in Figure 1, the outer Bowden cable sheath was constrained by the adjustable stop, which could be tightened to create pretension of the inner cable. The inner cable was connected to the grooved pulleys which were mounted on output shaft of the actuator and the knee joint, respectively. The thigh and shank brackets made of light-weight ABS plastics were connected together by the rotational knee joint of the exoskeleton for the swing motion in the saggital plane. Flexible Velcro straps were used for the fixation of the associated brackets to the lower limbs of the user. To provide feedback of the angular displacement of the user’s knee joint movement, a knee-angular sensor was installed on the knee axle of the exoskeleton.

Approximate weights and sizes of the major exoskeleton components are listed in Table 1 below. Here, the weight of waist bracket assembly (including the actuator module, the battery pack and controller box) was around 2160 g, while the weights of the exoskeleton thigh and shank brackets were 485 g and 418 g, respectively. With the proposed design, most of the weight is carried by the back straps and the pelvis of the human user, where only less than 1/3 of the weight is carried by the lower limbs of user. Furthermore, the lengths of both the thigh and shank brackets as well as back straps are adjustable for users with different body sizes. The optimized weight distribution and adjustable design provide good user comfort and portability of the soft exoskeleton.

### 2.2. Actuator Design

**Figure 2:**
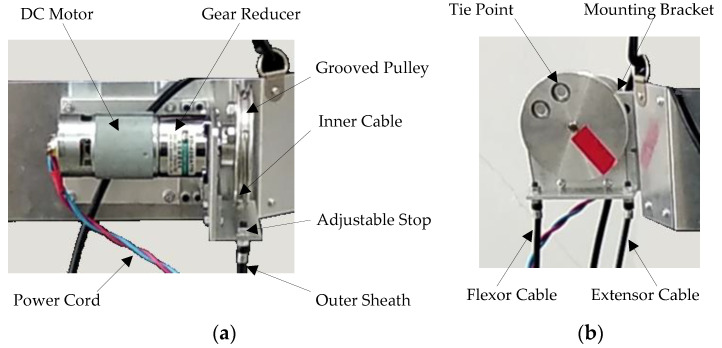
Actuator module used in the soft knee exoskeleton. (**a**) Front view of the actuator assembly. (**b**) Side view of the actuator assembly.

The Bowden transmission cables are driven by a custom-designed actuator module, which consists of a DC motor with a gear reducer (XD-42GA775-24V-25W, 50 round × min^−1^, gear ratio: 100:1, maximum torque: 0.917 N × m), a grooved pulley, cable tie points, and amounting bracket, as shown in Figure 2 below. This DC motor is a low-cost and relatively light-weight (540 g) motor without built-in encoders, as opposed to the expensive servo motors. It is useful for the portable design of the soft knee exoskeleton, as the motor can be mounted directly on the waist brace of the user.

When the DC motor rotates, the grooved pulley pulls the inner Bowden cables in two directions to actuate the knee extension and flexion, respectively. Adjustable stops are integrated for the pretension of inner cables, which could reduce the amount of backlash in the Bowden cables to a certain extent. For instance, the backlash could be reduced to around ±3 mm after adjustment, when a 1 m-long Bowden cable is used with a 90° bending angle and a 2.5 mm inner diameter. The amount of backlash of the gear reducer, however, cannot be reduced by the adjustable stops. Appropriate pretension is required, as very large pretension will induce high friction between the inner cable and outer sheath, causing a significant amount of power loss during the Bowden cable transmission. While this custom-designed actuator is of a compact structure, due to limited output power (25 W), it is unable to fully reject the frictional disturbances in the Bowden transmission cable during operation, using conventional control methods. This issue is analyzed in detail in the following sections.

## 3. Mathematical Modeling of the Exoskeleton System

### 3.1. Nonlinear Friction in the Bowden Cable

**Figure 3:**
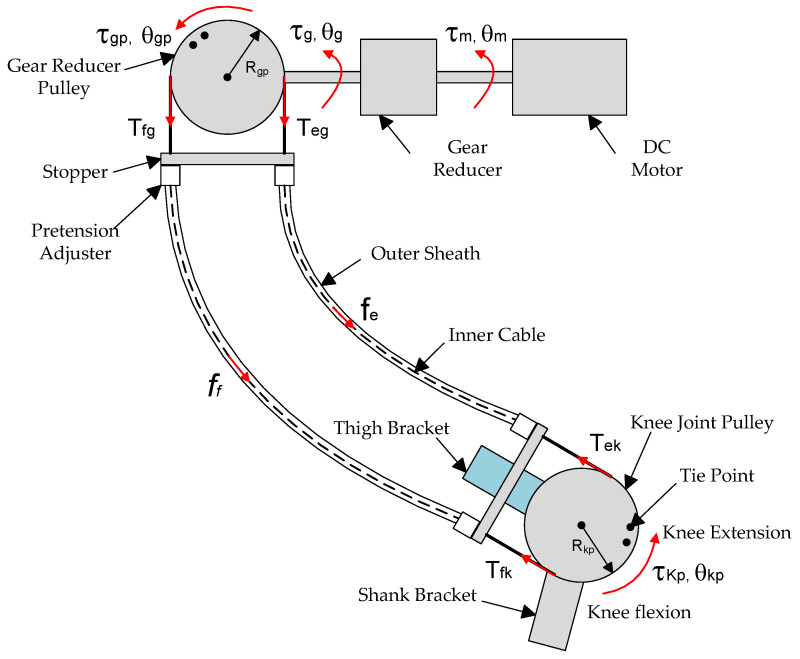
The Bowden cable transmission system for the soft knee exoskeleton. Backlash and friction exist in both the Bowden cables and gear reducer of the DC motor.

The Bowden cable transmission system for the soft knee exoskeleton is illustrated in Figure 3. The gear reducer lowers the rotational speed of DC motor and amplifies its output torque, which drives the gear-reducer pulley. As the inner cables are pulled by the gear-reducer pulley and slide inside the outer sheath, nonlinear friction is produced between the contacting interfaces. The friction results in the difference between the output and the input forces of the Bowden transmission cable.

To illustrate the nonlinear frictional effect quantitatively, a frictional model is needed. In this paper, the Coulomb friction between the inner cable and outer sheath was assumed, and the inertia of the inner cable was neglected. The input–output relationship of the inner cable’s tension can be described by the following equations [35,36]

(1) $$T_{out}=T_{in}\exp\left(-\mu \mathrm{sgn}\left(\dot{s}\right)\phi \left(L\right)\right)+T_{0}(\dot{s})$$

(2) $$\phi \left(L\right)=\int_{0}^{L}\kappa \left(\lambda \right)d\lambda$$

where $T_{in}$ and $T_{out}$ are the tensions of the inner cable at the input and output ends, respectively, $T_{0}$ is the inherent cable friction when the bending angle $\phi \left(L\right)$ is zero, μ is the frictional coefficient between the inner cord and the outer sheath, $sgn\left(\dot{s}\right)$ is the pulling direction of the inner cord, L is the length of the cable, $\kappa \left(\lambda \right)$ is the curvature, and $\phi \left(L\right)$ is the total bending angle of the Bowden cable.

Moreover, the input–output relationship of the tendon displacement (i.e., the variation of the tendon length) can be modeled as

(3) $$S_{out}=S_{in}+\delta \left(s\right)-\mathrm{sgn}\left(\dot{s}\right)B_{c}$$

(4) $$\delta \left(s\right)=\left(\int_{0}^{s}\frac{T\left(\lambda \right)}{EA}d\lambda \right)$$

where $S_{in}$ and $S_{out}$ are the displacements of the input and output ends of the inner cable, respectively, $\delta \left(s\right)$ is the elongation of the inner cord, $B_{c}$ is the backlash of the Bowden cable, E is the Young’s modulus of the tendon, and A is its cross-sectional area.

**Figure 4:**
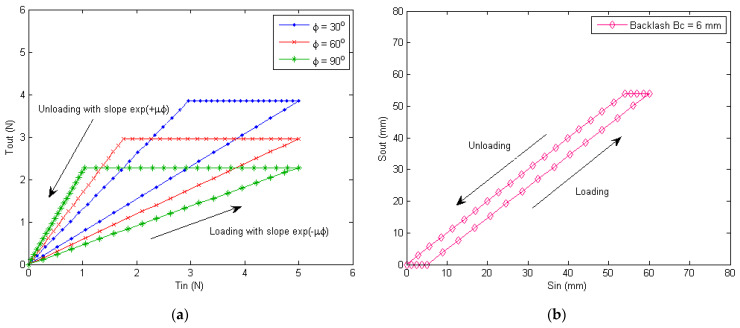
Loading and unloading curves for the Bowden cable friction and backlash. (**a**) Simulated relationship of the input and output tensions of the Bowden cable, for friction coefficient μ=0.5, at different bending angles ϕ. (**b**) Simulated relationship of the input and output displacements of the Bowden cable, with a cable backlash of $B_{c}=6\;\mathrm{mm}$.

The simulated input–output relationship for cable tensions and displacements can be obtained based on Equations (1)–(4), as shown in Figure 4 below. Figure 4a shows the loading and unloading characteristics of the cable tensions when the friction coefficient is μ=0.5 and the bending angles are $\phi =30^{o},\;60^{o},\;90^{o}$, respectively. The plot shows the difference between loading and unloading process, and there is a region where the output tension is unchanged. Such characteristics will lead to frictional hysteresis, creating larger tracking errors when controlling the soft exoskeleton. Figure 4b plots the loading and unloading profile when a backlash of the Bowden cable $B_{c}=6\;\mathrm{mm}$ exists. The backlash of the Bowden cable and gear reducer together with the cable friction contribute to the complex nonlinear hysteresis of the knee joint motion.

### 3.2. Dynamics of the Soft Knee Exoskeleton System

The dynamics of the soft knee exoskeleton system involve the motions of the exoskeleton knee joint, the gear-reducer pulley, and the DC motor, all which contribute to the degrees of freedom of the system. The most important degree of freedom is the knee joint rotation, which was the focus of control in this study. The rotation of the gear-reducer pulley and motor spindle are additional degrees of freedom but are of less concern in the controller design. Moreover, the bending angles of the flexion and extension Bowden cables as well as the human body motion also affect the dynamical performance, but they are not controllable and are treated as disturbances.

The following analysis formulates the mathematical equations for these motions. Here, certain approximations and simplifications were made, which include the assumption of Coulomb friction in the Bowden transmission cable and linear viscous friction in the rotational axles (e.g., motor spindles, knee joint bearing, etc.). The goal was to derive the dynamical relationship between the output torque of the DC motor and the angular response of the knee joint.

The equation of motion for the soft exoskeleton knee joint can be written as

(5) $$J_{kp}{\ddot{\theta }}_{kp}(t)+c_{kp}{\dot{\theta }}_{kp}(t)+h(\theta ,t)+g(\theta ,t)=\tau_{kp}(t)$$

where $J_{kp}$ is the equivalent moment of inertia of the knee pulley (including both the exoskeleton and human),$c_{kp}$ is the viscous friction coefficient of the exoskeleton knee joint bearing, $h\left(\theta ,t\right)$ is the disturbance torque by the human body, $g\left(\theta ,t\right)$ is the gravitational torque, and $\tau_{kp}$ is the total actuation torque applied by the Bowden cables on the knee pulley.

The actuation torque $\tau_{kp}$ on the exoskeleton knee pulley and $\tau_{gp}$ on the gear-reducer pulley are formulated below, which are related to the Bowden cable tensions and radii of pulleys

(6) $$\tau_{kp}(t)=\left(T_{ek}(t)-T_{fk}(t)\right)R_{kp}$$

(7) $$\tau_{gp}(t)=\left(T_{eg}(t)-T_{fg}(t)\right)R_{gp}$$

where $T_{ek}\left(t\right)$ and $T_{fk}\left(t\right)$ are the extensor and flexor cable tensions on the knee pulley respectively, $T_{eg}\left(t\right)$ and $T_{fg}\left(t\right)$ are the extensor and flexor cable tensions on the gear-reducer pulley respectively, and $R_{kp}$ and $R_{gp}$ are radii of the knee and gear-reducer pulleys respectively.

Due to friction, the tensions on each side of the Bowden cable are different for both the extension and flexion cables. The detailed relationship can be derived based on Equation (1), namely

(8) $$\begin{array}{l} T_{ek}=T_{eg}\exp\left(-\mu \mathrm{sgn}({\dot{\theta }}_{gp}+{\dot{\theta }}_{kp})\phi_{e}(t)\right)-f_{e0}({\dot{\theta }}_{gp},{\dot{\theta }}_{kp})\\ \;\;=T_{eg}-f_{e}(\phi_{e}(t),{\dot{\theta }}_{gp},{\dot{\theta }}_{kp})-f_{e0}({\dot{\theta }}_{gp},{\dot{\theta }}_{kp}) \end{array}$$

(9) $$\begin{array}{l} T_{fk}=T_{fg}\exp\left(+\mu \mathrm{sgn}({\dot{\theta }}_{gp}+{\dot{\theta }}_{kp})\phi_{f}(t)\right)-f_{f0}({\dot{\theta }}_{gp},{\dot{\theta }}_{kp})\\ \;\;=T_{fg}-f_{f}(\phi_{f}(t),{\dot{\theta }}_{gp},{\dot{\theta }}_{kp})-f_{f0}({\dot{\theta }}_{gp},{\dot{\theta }}_{kp}) \end{array}$$

where

(10) $$f_{e}(\phi_{e}(t),{\dot{\theta }}_{gp},{\dot{\theta }}_{kp})=T_{eg}\left[1-\exp\left(-\mu \mathrm{sgn}({\dot{\theta }}_{gp}+{\dot{\theta }}_{kp})\phi_{e}(t)\right)\right]$$

(11) $$f_{f}(\phi_{f}(t),{\dot{\theta }}_{gp},{\dot{\theta }}_{kp})=T_{fg}\left[1-\exp\left(-\mu \mathrm{sgn}({\dot{\theta }}_{gp}+{\dot{\theta }}_{kp})\phi_{f}(t)\right)\right]$$

here, $\phi_{e}\left(t\right)$ and $\phi_{f}\left(t\right)$ are the total bending angles of the extension and flexion Bowden cables respectively, ${\dot{\theta }}_{gp}\left(t\right)$ and ${\dot{\theta }}_{kp}\left(t\right)$ are the angular velocity of the gear-reducer pulley and knee joint pulley respectively and their sum is used to determine the direction of the average cable velocity, $f_{e}\left(\phi_{e}\left(t\right)\right)$ and $f_{f}\left(\phi_{f}\left(t\right)\right)$ are friction caused by the bending angles in the extension and flexor cables respectively [37], and $f_{e0}$ and $f_{f0}$ are inherent cable friction when bending angles are zero in the extension and flexor cables respectively.

The dynamical model of the DC motor can be formulated as

(12) $$u(t)-K_{e}{\dot{\theta }}_{m}(t)=L_{a}{\dot{i}}_{m}(t)+R_{a}i_{m}(t)$$

(13) $$\tau_{m}(t)=K_{T}i_{m}(t)$$

(14) $$J_{m}{\ddot{\theta }}_{m}(t)+c_{m}{\dot{\theta }}_{m}(t)=\tau_{m}(t)$$

where $u\left(t\right)$ is the motor control voltage, $K_{e}$ is the motor back-EMF constant, $L_{a}$ and $R_{a}$ are the armature inductance and resistance respectively, $i_{m}\left(t\right)$ is the armature current, $\tau_{m}\left(t\right)$ is the motor torque, $\theta_{m}\left(t\right)$ is the motor angle, $K_{T}$ is the motor torque constant, and $J_{m}$ and $c_{m}$ are the moment of inertia and viscous friction coefficient of the motor spindle respectively.

The gear reducer lowers the output velocity of the DC motor and amplifies the motor’s torque, which can be described by

(15) $$\tau_{g}(t)=\alpha \eta ({\dot{\theta }}_{m})\tau_{m}(t)=\alpha \tau_{m}(t)-\mathrm{sgn}({\dot{\theta }}_{m})\tau_{fg}(t)$$

(16) $$\theta_{g}(t)=\theta_{m}(t)/\alpha +\mathrm{sgn}({\dot{\theta }}_{m})B_{g}$$

$$\tau_{fg}(t)=c_{gp}{\dot{\theta }}_{gp}$$

where $\tau_{g}\left(t\right)$ and $\tau_{m}\left(t\right)$ are the output torques of the gear reducer and DC motor respectively, $\theta_{g}\left(t\right)$ and $\theta_{m}\left(t\right)$ are the output angles of the gear reducer and DC motor respectively, α is the gear ratio, $\eta ({\dot{\theta }}_{m})$ is the efficiency of the gear reducer, $\tau_{fg}$ is the equivalent frictional torque in the gear reducer, $B_{g}$ is the gear reducer’s backlash, and $c_{gp}$ is the viscous friction coefficient of the gear reducer.

For the dynamical motion of the gear reducer pulley, the following holds

(17) $$J_{gp}{\ddot{\theta }}_{gp}=\tau_{g}(t)-\tau_{gp}(t)$$

(18) $$\theta_{gp}(t)=\theta_{g}(t)$$

where $J_{gp}$ is the equivalent moment of inertia of the gear-reducer pulley.

Combining Equations (5)–(18) and assuming zero pretentions of the extension and flexion cables, the relationship between the output torque of the DC motor and the angular displacement of exoskeleton knee joint can be written as

(19) $${\tilde{J}}_{kp}{\ddot{\theta }}_{kp}(t)+{\tilde{c}}_{kp}{\dot{\theta }}_{kp}(t)+\left({\tilde{\tau }}_{f}(\phi (t),{\dot{\theta }}_{gp},{\dot{\theta }}_{kp})-{\tilde{\tau }}_{f0}({\dot{\theta }}_{kp})\right)+h(\theta ,t)+g(\theta ,t)=\alpha \left(R_{kp}/R_{gp}\right)\tau_{m}(t)$$

where

(20) $${\tilde{J}}_{kp}=\left(\alpha^{2}J_{m}+J_{gp}\right){\left(R_{kp}/R_{gp}\right)}^{2}+J_{kp}$$

(21) $${\tilde{c}}_{kp}=\left(\alpha c_{m}+c_{gp}\right)\left(R_{kp}/R_{gp}\right)+c_{kp}$$

(22) $${\tilde{\tau }}_{f}(\phi (t),{\dot{\theta }}_{gp},{\dot{\theta }}_{kp})\approx \alpha \eta ({\dot{\theta }}_{m})\left[1-\exp\left(-\mu \mathrm{sgn}({\dot{\theta }}_{gp}+{\dot{\theta }}_{kp})\phi_{e}(t)\right)\right]\left(R_{kp}/R_{gp}\right)\tau_{m}(t)$$

(23) $${\tilde{\tau }}_{f0}({\dot{\theta }}_{gp},{\dot{\theta }}_{kp})=\left(f_{e0}({\dot{\theta }}_{gp},{\dot{\theta }}_{kp})-f_{f0}({\dot{\theta }}_{gp},{\dot{\theta }}_{kp})\right)R_{kp}$$

here, ${\tilde{J}}_{gp}$ and ${\tilde{c}}_{gp}$ are the equivalent overall moment of inertia and viscous friction coefficient on the knee joint respectively, ${\tilde{\tau }}_{f}(\phi \left(t\right),{\dot{\theta }}_{kp})$ is the overall frictional torque caused by the bending angle of the Bowden transmission cable (in quasi-static condition), and ${\tilde{\tau }}_{f0}({\dot{\theta }}_{kp})$ is the overall inherent frictional torque when bending angle is zero.

As Equation (19) shows, the human–exoskeleton system can be modeled as a second-order system with viscous damping, nonlinear cable frictional disturbances, human motion disturbance, and gravitational disturbance. The nonlinear cable friction causes a significant amount of backlash hysteresis in the angular displacement output of the soft knee exoskeleton when conventional control methods are used without appropriate compensation. The following section focuses on the controller design for this particular system, which aims to address the issue of frictional backlash.

## 4. Controller Design

**Figure 5:**
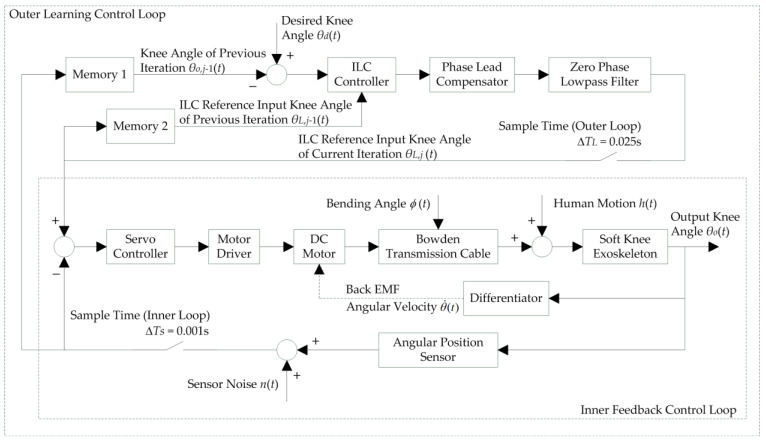
Controller design with an inner stabilizing feedback loop and outer phase-lead ILC loop. The inner loop stabilizes the position control of the soft exoskeleton knee joint, sampling at 1 kHz. The outer loop updates the ILC reference input knee angle iteratively at 40 Hz for the tracking error to converge to zero (or a very small level).

For successful and precise operation of the soft knee exoskeleton, a good design of the controller is crucial, which includes low tracking error and robust stability to model uncertainties. In particular, a fast response with low overshoot is required for the Bowden-cable-actuated system, which has a considerable amount of delay and hysteresis due to cable friction and gear-reducer backlash. Moreover, monotonic decay (convergence) of tracking error is required to prevent injuries to the user during use. The overall design of the control system for the soft knee exoskeleton is presented in Figure 5 below, which consists of an inner-loop feedback controller and an outer-loop iterative learning controller.

The inner loop is a lower-level servo control with a high sampling rate at 1 kHz ($\Delta T_{S}=0.001\;s$). The angular displacement sensor reads the angular position $\theta_{O}\left(t\right)$ of the knee joint as a feedback signal which is compared with the ILCreference input knee angle signal $\theta_{L}\left(t\right)$ for the inner-loop servo controller to process. Here, the angular velocity ${\dot{\theta }}_{O}\left(t\right)$ is obtained by a basic differentiation of the angular displacement $\theta_{O}\left(t\right)$. The differentiator works well in this study with proper shielding of the wirings and sufficient allocation of A/D conversion time. More advanced methods (such as Kalman Filtering [38]) offer better estimate of velocity in the presence of noise. However, they have not been implemented due to the finite computation power of the microprocessor and limited knowledge of the model [39]. The control parameters of the inner-loop servo controller were tuned to ensure closed-loop stability of the position control for the exoskeleton knee angle. The motor driver amplifies the output signal of the servo controller and drives the DC motor. Additionally, Bowden cables transmit the rotational motion of the DC motor to the exoskeleton knee joint. The mission of the inner-loop control is to make the output knee angle $\theta_{O}\left(t\right)$ as close as possible to the reference input knee angle $\theta_{L}\left(t\right)$ of the inner loop. However, due to Bowden cable friction and disturbances such as human motion $h\left(t\right)$ and gravitational forces $g\left(\theta ,t\right)$, the position-tracking performance of the inner-loop controller alone is poor.

For this problem, an outer learning control loop was introduced to compensate for the frictional disturbances of the Bowden transmission cable. In typical exoskeleton applications such as rehabilitation training and walking assistance, repetitive movements of the human body and exoskeleton are involved, which inspires a learning-based approach. The proposed learning-based repetitive control belongs to the more general internal model principle (IMP). It is a particular case of the IMP for tracking periodic references or attenuating periodic disturbances in closed-loop control [40,41]. Basically, the outer loop creates a process that simulates the response of the system in order to estimate the outcome of a system disturbance. Intuitively, the model of the dynamic structure of the environment is created in the closed loop system using the proposed approach [42,43]. For the particular exoskeleton system, the outer learning control loop learns the history of output knee joint angle $\theta_{O,j-1}\left(t\right)$ and input reference $\theta_{L,j-1}\left(t\right)$ for the inner loop in the previous repetition j−1.Some learning laws and filtering are then applied, which update the input reference $\theta_{L,j}\left(t\right)$ for the inner loop in the current repetition j. The refresh rate of the outer learning loop is at 40 Hz ($\Delta T_{L}=0.025\;s)$. When properly designed, the iterative adjustment of the input reference $\theta_{L,j-1}\left(t\right)$ for the inner loop could make the tracking error ($\theta_{d}\left(t\right)-\theta_{O}\left(t\right)$) converge to zero (or a very small level) as the number of iterations increases.

### 4.1. Design of Inner Loop Digital Servo Controller

**Figure 6:**
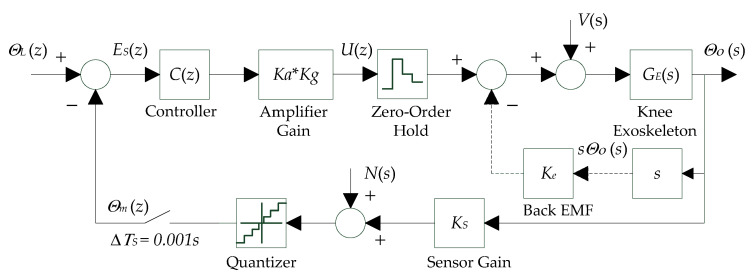
Block diagram of the inner-loop digital servo controller.

The block diagram of the inner-loop digital servo control system is illustrated in Figure 6 below, where $\Theta_{L}\left(z\right)$ is the reference input knee angle and $\Theta_{O}\left(z\right)$ is the output knee angle. The goal of the inner-loop digital servo controller is to stabilize the angular output of the soft knee exoskeleton $G_{E}\left(s\right)$, by proper design of the controller $C\left(z\right)$.To ensure a fast response and dynamical stability, a high sampling rate of 1 kHz ($\Delta T_{S}=0.001\;s)$ wasused for the inner-loop digital servo controller.

The soft knee exoskeleton model can be obtained from Equation (19), and its continuous time transfer function $G_{E}\left(s\right)$ can be written as

(24) $$G_{E}(s)=\frac{1}{{\tilde{J}}_{kp}s^{2}+{\tilde{c}}_{kp}s}$$

where s is the Laplace operator, and ${\tilde{J}}_{kp}$ and ${\tilde{c}}_{kp}$ are the equivalent moment of inertia and viscous friction coefficient on the knee joint, respectively.

Based on the digital sampling of the A/D converter (quantizer) and zero-order hold of the D/A converter (motor amplifier),the discrete-time model of the soft knee exoskeleton $G_{E}\left(z\right)$ can be written in the following form

(25) $$G_{E}(z)=\frac{\left(\xi -1+e^{-\xi }\right)z+\left(1-e^{-\xi }-\xi e^{-\xi }\right)}{\xi {\tilde{c}}_{kp}\left(z-1\right)\left(z-e^{-\xi }\right)}=\frac{a_{1}z+a_{0}}{z^{2}+b_{1}z+b_{0}}$$

(26) $$\xi =\frac{{\tilde{c}}_{kp}}{{\tilde{J}}_{kp}}\Delta T_{s}$$

where the parameters $a_{1}$, $a_{0}$, $b_{1}$, $b_{0}$, ξ depend on the exoskeleton parameters, digital sampling time $\Delta T_{S}$, and zero-order hold being used, and z is the z-transform operator.

The Bowden cable friction $F\left(z\right)$, human motions $H\left(z\right)$,and gravitational forces $G\left(z\right)$ are all treated as disturbances, represented by the term $V\left(z\right)$ in the discrete-time domain, namely

(27) V(z)=F(z)+H(z)+G(z)

In this study, a digital servo controller $C\left(z\right)$ of a proportional-derivative (PD) type wasused, and its discrete-time transfer function can be expressed as

(28) $$C(z)=k_{p}+k_{d}\left(\frac{z-1}{z}\right)$$

where $k_{p}$ and $k_{d}$ are the proportional and derivative gains.

By mathematical manipulation of the block diagram in Figure 6 above, the following equations hold

(29) $$\left(K_{a}K_{g}\left(\Theta_{L}(z)-\Theta_{m}(z)\right)C(z)+V(z)\right)G_{E}(z)=\Theta_{O}(z)$$

(30) $$\Theta_{O}(z)+N(z)=\Theta_{m}(z)$$

where $K_{a}$ and $K_{g}$ are the motor amplifier gain and gear ratio respectively, $\Theta_{m}\left(z\right)$ is the measured knee angle, $N\left(z\right)$ is the measurement noise and unity sensor gain, and $K_{S}$ is assumed.

Therefore, the transfer functions from the reference input $\Theta_{L}\left(z\right)$, disturbance $V\left(z\right)$ and measurement noise $N\left(z\right)\;$ to the output knee angle $\Theta_{O}\left(z\right)$ can be obtained from Equations (29) and (30) as follows

(31) $$\frac{\Theta_{O}(z)}{\Theta_{L}(z)}=\frac{K_{a}K_{g}C(z)G_{E}(z)}{1+K_{a}K_{g}C(z)G_{E}(z)}$$

(32) $$\frac{\Theta_{O}(z)}{V(z)}=\frac{G_{E}(z)}{1+K_{a}K_{g}C(z)G_{E}(z)}$$

(33) $$\frac{\Theta_{O}(z)}{N(z)}=\frac{-K_{a}K_{g}C(z)G_{E}(z)}{1+K_{a}K_{g}C(z)G_{E}(z)}$$

Combining Equations (25), (28), and (31)yields the equivalent closed-loop transfer function $G_{IL}\left(z\right)$ of the inner loop

(34) $$G_{IL}(z)=\frac{K_{a}K_{g}\left(\left(k_{p}+k_{d}\right)z-k_{d}\right)\left(a_{1}z+a_{0}\right)}{z^{3}+\left(b_{1}+a_{1}K_{a}K_{g}\left(k_{p}+k_{d}\right)\right)z^{2}+\left(b_{0}+a_{0}\left(k_{p}+k_{d}\right)-a_{1}k_{d}\right)z-a_{0}K_{a}K_{g}k_{d}}$$

The servo error of the inner digital feedback loop is defined as

(35) $$E_{S}(z)=\Theta_{L}(z)-\Theta_{m}(z)$$

And the characteristic equation of the servo error $E_{S}\left(z\right)$ can be obtained based on the numerator of Equation (34)

(36) $$z^{3}+\left(b_{1}+a_{1}K_{a}K_{g}\left(k_{p}+k_{d}\right)\right)z^{2}+\left(b_{0}+a_{0}\left(k_{p}+k_{d}\right)-a_{1}k_{d}\right)z-a_{0}K_{a}K_{g}k_{d}=0$$

The dynamic performance and stability of the inner loop can be changed by adjusting $k_{p}$ and $k_{d}$. Assuming the desired closed-loop poles of the servo error’s characteristic equation are $p_{1}$, $p_{2}$, and $p_{3}$, the following holds

(37) $$\left(z-p_{1}\right)\left(z-p_{2}\right)\left(z-p_{3}\right)=0$$

By equating the corresponding coefficients in Equations (36) and (37), the relationship between the control gains and desired closed-loop poles can be established as

(38) $$\left\{\begin{array}{l} b_{1}+a_{1}K_{a}K_{g}\left(k_{p}+k_{d}\right)=p_{1}+p_{2}+p_{3}\\ b_{0}+a_{0}\left(k_{p}+k_{d}\right)-a_{1}k_{d}=p_{1}p_{2}+p_{2}p_{3}+p_{3}p_{1}\\ a_{0}K_{a}K_{g}k_{d}=p_{1}p_{2}p_{3} \end{array}\right.$$

For the discrete-time stability of the inner loop servo control, $k_{p}$ and $k_{d}$ can be selected such that the eigenvalues of Equation (36) are all within the circle with radius ρ, that is

(39) $$\left|p_{1}\right|<\rho ,\;\left|p_{2}\right|<\rho ,\;\left|p_{3}\right|<\rho \;(\rho \leq 1)$$

where ρ=1 for basic stability and ρ<1 for faster settling time. To avoid overshoot, the closed-loop poles $p_{1}$, $p_{2}$, and $p_{3}$ should all be real numbers.

**Figure 7:**
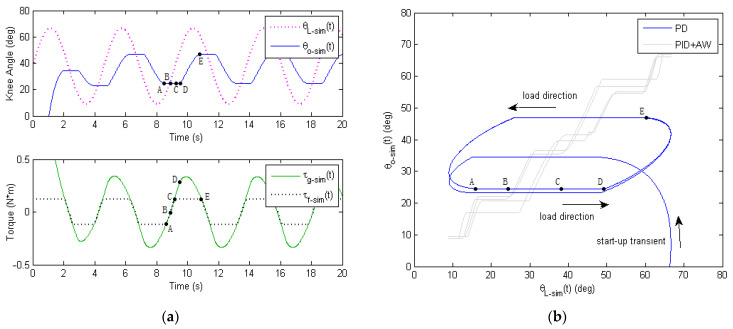
Position tracking performance of the inner-loop digital servo controller with Bowden cable friction (μ=0.5, $\phi =90^{o}$) and gear-reducer backlash of $B_{g}=\pm 2^{o}$. (**a**) Simulated sinusoidal response of the soft knee exoskeleton. (**b**) Simulated relationship of the reference input $\theta_{L-sim}\left(t\right)$ and output displacements $\theta_{O-sim}\left(t\right)$ of the exoskeleton knee joint.

Based on the inner-loop digital controller design above, a Matlab Simulink simulation was conducted to study the sinusoidal response of the soft knee exoskeleton system. The simulated results are shown in Figure 7 below, where the PD controller gains are $k_{p}=4$, $k_{d}=0.5$, the amplifier gain (with motor torque constant) is $K_{a}K_{g}=0.225\;\mathrm{Nm}$, the equivalent moment of inertia on knee pulley is ${\tilde{J}}_{kp}=0.01{\;\mathrm{kgm}}^{2}$, and the viscous coefficientis ${\tilde{c}}_{kp}=0.7\;\mathrm{Nms}/\mathrm{rad}$. Moreover, to match the experimental results, frictional coefficient μ=0.5 and bending angle $\phi =90^{o}$ were used for the Bowden cable, and gear-reducer backlash was $B_{g}=\pm 2^{o}$ based on hardware measurement.

The upper plot in Figure 7a shows the position tracking performance of the inner-loop digital servo controller, and the lower plot in Figure 7a shows the time history of the gear-reducer output torque $\tau_{g-sim}$ and equivalent frictional torque $\tau_{f-sim}$. As seen from Figure 7a, there was a significant amount of backlash and phase delay for the output knee angle $\theta_{O-sim}\left(t\right)$ compared with the reference input $\theta_{L-sim}\left(t\right)$. Figure 7b shows the input–output relationship by an X–Y plot, which further illustrates the issue of frictional hysteresis and backlash. At point A, the output torque of gear reducer started to fall below the maximum frictional torque and the exoskeleton system slowed down and stop edits rotation. From point A to point B, the Bowden cable provides a static friction which cancels the output torque of the gear reducer. From point B to point C, the output torque of gear reducer switched sign, and the static Bowden cable friction also switched sign. From point C to point D, the output torque of the gear reducer surpassed the maximum frictional torque and the motor spindle started to rotate. However, due to the gear-reducer backlash, the output knee angle of the exoskeleton still remained unchanged, until point D was reached. Therefore, the exoskeleton system kept stationary from point A to point C, and static friction was applied against the output torque of the gear reducer. From point D to point E, the knee exoskeleton started rotating again, and dynamic friction was applied against the output torque of gear reducer.

The introduction of an integral term with anti-windup (AW) could help improve the tracking performance to some extent, as seen from the gray curve, Figure 7b. However, the tracking error of PID +AW control is still not small enough (RMSE ~3.254 deg). In addition, the output response of the PID + AW control is quite choppy due to the nonlinear Bowden cable friction and backlash when a more aggressive Ki term is used. While advanced PID + AW methods have been developed previously, the mathematical modeling involved is quite complicated [44,45], which is beyond the focus of this paper (i.e., the design of ILC). Furthermore, with the extra Ki and AW parameters, the PID + AW require more intensive tuning, especially when the operational conditions change (e.g., different human weight, different ground slope, etc). As a result, the PID + AW alone are still undesired for the safe and smooth operation of the soft exoskeleton robot.

While the analysis above is based on the theoretical simulation with certain simplifications made, the simulated curves offer useful insight into the physical causes for the frictional hysteresis and backlash. Due to the highly nonlinear and time-varying characteristic of the friction and backlash, the PD-type (or PID + AW) inner-loop digital servo controller alone is unable to track the reference curve accurately. Additionally, an outer learning control loop is needed, which is discussed below.

### 4.2. Design of Outer Loop Iterative Learning Controller

**Figure 8:**
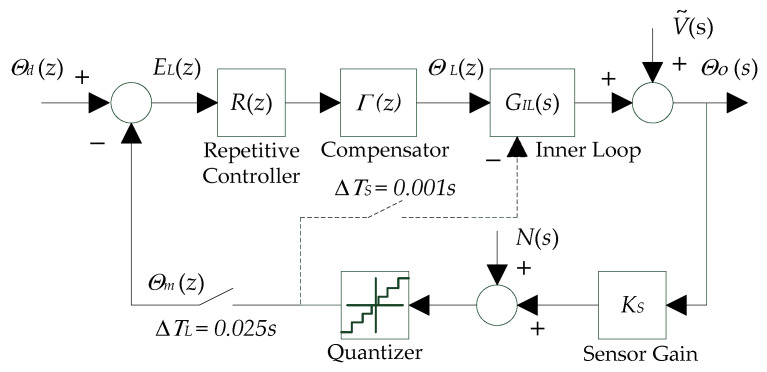
Block diagram of the outer-loop learning-based repetitive controller.

The block diagram of the outer-loop learning-based repetitive control system is illustrated in Figure 8 below, where $\Theta_{d}\left(z\right)$ is the desired knee angle for the outer loop, $\Theta_{L}\left(z\right)$ is the reference input knee angle for the inner loop, and $\Theta_{O}\left(z\right)$ is the output knee angle. The goal of the outer-loop learning-based repetitive controller is to adjust the reference input knee angle $\Theta_{L}\left(z\right)$ each cycle so that the output of the soft knee exoskeleton $\Theta_{O}\left(z\right)$ will approach the desired knee angle $\Theta_{d}\left(z\right)$ as the number of iterations increases. The design of the repetitive controller $R\left(z\right)$ and compensator $\Gamma \left(z\right)$ is crucial for the fast convergence and good transients (i.e., monotonic convergence) of learning. The speed of convergence is important for the efficient operation of soft exoskeleton, where the periodic tracking error could be reduced in as few iterations as possible through learning. Furthermore, the safe and smooth operation of the soft exoskeleton requires monotonic convergence of tracking error, so that any potential injury caused by large error transients (i.e., error in intermediate iterations) to the human user could be avoided. Here, a lower sampling rate of 40 Hz ($\Delta T_{L}=0.025\;s)$ was used for the outer-loop learning-based repetitive controller, which further helped stabilize the learning and provided adequate settling for the inner-loop PD controller.

The proposed learning-based repetitive controller can be formulated as follows [46]:

(40) $$\theta_{L,j}\left(k\right)=\theta_{L,j-1}\left(k\right)+\sum_{i=i_{0}}^{i=i_{f}}\varphi \left(i\right)e_{L,j-1}\left(k+\gamma +i\right)$$

where $\theta_{L,j}\left(k\right)$ and $\theta_{L,j}\left(k\right)$ are the reference input knee angles at time step k of iteration j and $\left(j-1\right)$ respectively, $e_{L,j-1}\left(k\right)=\theta_{d}\left(k\right)-\theta_{m,j-1}\left(k\right)$ is the tracking error of the outer learning loop of iteration $\left(j-1\right)$, $\varphi \left(i\right)$ is the coefficient of learning gain for $e_{L,j-1}\left(k+\gamma +i\right)$, γ is the parameter for phase-lead compensation, and k=1, 2, 3,…, p with p being the total number of samples per iteration.

Note that time step k of the previous iteration $\left(j-1\right)$ is shifted by p time steps backward with respect to the current iteration j. Therefore, the following holds

(41) $$\theta_{L,j-1}\left(k\right)=\theta_{L,j}\left(k-p\right)$$

(42) $$e_{L,j-1}\left(k+\gamma +i\right)=e_{L,j}\left(k-p+\gamma +i\right)$$

By substituting Equations (41) and (42) into Equation (40), the z-transform of the learning-based repetitive control law in Equation (40) can be obtained as

(43) $$\Theta_{L}\left(z\right)=R\left(z\right)\Gamma \left(z\right)E_{L}\left(z\right)$$

where the compensator $\Gamma \left(z\right)$ is defined as

(44) $$\Gamma \left(z\right)=\varphi \left(i_{0}\right)z^{i_{0}}+\varphi \left(i_{0}+1\right)z^{i_{0}+1}+\cdot \cdot \cdot +\varphi \left(i_{f}-1\right)z^{i_{f}-1}+\varphi \left(i_{f}\right)z^{i_{f}}$$

and the repetitive controller is defined as

(45) $$R\left(z\right)=\frac{z^{\gamma }}{z^{p}-1}$$

Moreover, the block diagram in Figure 8 implies the following relationship

(46) $$\left\{\begin{array}{l} E_{L}\left(z\right)=\Theta_{d}\left(z\right)-\Theta_{m}\left(z\right)\\ \Theta_{L}\left(z\right)G_{IL}\left(z\right)+\tilde{V}\left(z\right)+N\left(z\right)=\Theta_{m}\left(z\right) \end{array}\right.$$

Combing Equations (43), (45), and (46) produces the dynamical equation for the error $E_{L}\left(z\right)$ of the outer learning loop as

(47) $$\begin{array}{l} \left[z^{\gamma }G_{IL-N}\left(z\right)\Gamma_{N}\left(z\right)+\left(z^{p}-1\right)G_{IL-D}\left(z\right)\Gamma_{D}\left(z\right)\right]E_{L}\left(z\right)\\ =\left[G_{IL-D}\left(z\right)\Gamma_{D}\left(z\right)\left(z^{p}-1\right)\right]\left[\Theta_{m}\left(z\right)-\tilde{V}\left(z\right)-N\left(z\right)\right] \end{array}$$

where $G_{IL-N}\left(z\right)$ and $G_{IL-D}\left(z\right)$ are the numerator and denominator of the equivalent inner loop z-transfer function $G_{IL}\left(z\right)$, and $\Gamma_{N}\left(z\right)$ and $\Gamma_{D}\left(z\right)$ are the numerator and denominator of the compensator z-transfer function $\Gamma \left(z\right)$. And the characteristic equation for the error dynamics is

(48) $$\left[z^{\gamma }G_{IL-N}\left(z\right)\Gamma_{N}\left(z\right)+\left(z^{p}-1\right)G_{IL-D}\left(z\right)\Gamma_{D}\left(z\right)\right]E_{L}\left(z\right)=0$$

The stability of the outer learning loop requires all the roots of the characteristic Equation (48) to be inside the unit circle. However, the high order of the characteristic polynomial produced by the number of samples p per iteration in Equation (48) makes it impractical to use this condition directly to determine stability. Methods such as finding the actual roots, Jury test, or Routh test with bilinear transformation would not work well in this case. Another way is to use the departure angle criteria of the root locus plot to determine stability. However, this method works only with sufficiently small learning gains [47]. This paper used the stability theory based on frequency domain analysis, namely the Nyquist stability theory.

Rearranging Equation (48) yields

(49) $$z^{p}E_{L}\left(z\right)=\left[1-z^{\gamma }\Gamma \left(z\right)G_{IL}\left(z\right)\right]E_{L}\left(z\right)$$

where $E\left(z\right)$ is the z-transformation of error at repetition $\left(j-1\right)$, and $z^{p}E\left(z\right)$ is the z-transform of the next repetition j. Then, the frequency transfer function from one repetition to the next can be obtained based on Equation (49). Assuming the settling time of the inner loop is short compared with the period of the knee angle signal $\theta_{O}\left(t\right)$, the condition for the monotonic decay in the outer learning loop is

(50) $$\left|1-e^{i\omega \Delta T_{L}\gamma }\Gamma \left(e^{i\omega \Delta T_{L}}\right)G_{IL}\left(e^{i\omega \Delta T_{L}}\right)\right|<1$$

for all ω up to the Nyquist frequency ($\omega_{N}=1/\left(2\Delta T_{L}\right)$).

**Figure 9:**
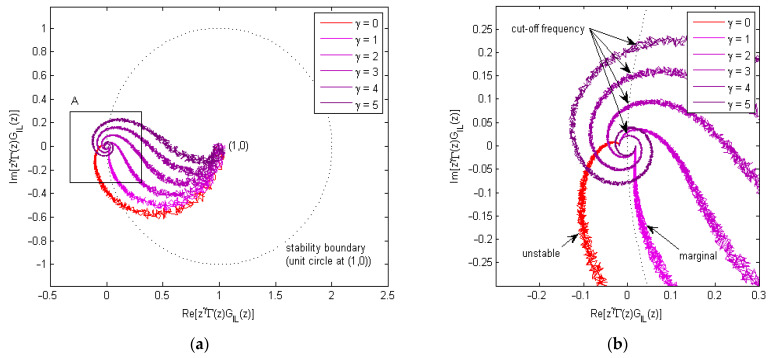
Stability plot using frequency domain analysis, with magnitude and phase noise added. And a basic P-type learning law with unity learning gain is used. (**a**)Nyquist plot of $z^{\gamma }\Gamma \left(z\right)G_{IL}\left(z\right)$, with the unit circle stability boundary shown. (**b**) Zoom-in plot of region A in Figure 9a, where the outer learning loop is unstable for γ=0, marginally stable for γ=1 and requires frequency cut-off for γ=2, 3, 4, 5.

The simulated plot based on the frequency-domain stability requirement in (41) is shown in Figure 9. To simulate the model uncertainty in frequency domain, noises were added to both the magnitude and phase of $z^{\gamma }\Gamma \left(z\right)G_{IL}\left(z\right)$. Here, a basic P-type learning law with unity learning gain was used, and the inner-loop nominal model is based on the simplified linear model of Equation (34) in Section 4.1. The monotonic decay condition in (41) requires the Nyquist plot of $z^{\gamma }\Gamma \left(z\right)G_{IL}\left(z\right)$ to be inside the unit circle at (1, 0). As seen from Figure 9b, the outer learning loop was unstable when there was no phase-lead compensation (γ=0) and marginally stable when γ=1. For higher values of γ, the learning can be stabilized using an additional cut-off filter to terminate the learning at higher frequencies [48].

**Figure 10:**
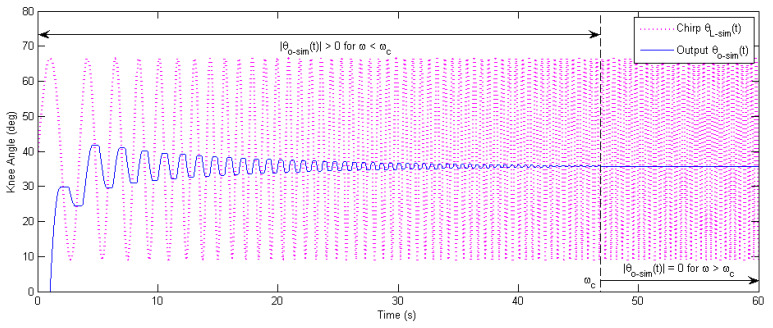
Sinusoidal response of a linear chirp signal based on the Matlab Simulink model. The frequency ω of the chirp input $\theta_{L-sim}\left(t\right)$ varies linearly from 0.2174 Hz to 2.6187 Hz for time t=0 s to 60 s. Due to friction and backlash in the Bowden cable and gear reducer, the simulated output knee angle $|\theta_{O-sim}\left(t\right)|=0$ for $\omega >\omega_{c}=2.0986\;\mathrm{Hz}$ (where other parameters are consistent with those in Figure 7).

Due to the friction and backlash of the Bowden transmission cable and gear reducer, the magnitude response of the real-world inner-loop model is actually zero when the excitation frequency ω is above a certain value $\omega_{c}$ (shown in Figure 10). Such a nonlinear effect acts as a physical cut-off filter, which helps stabilize the learning process for higher frequencies, even without a digital cut-off filter.

**Figure 11:**
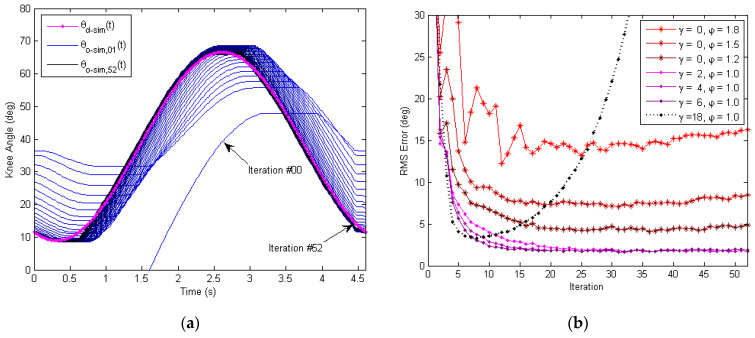
Time-domain simulation of the outer-loop learning-based repetitive controller. A P-type learning law with the phase-lead compensation is used. (**a**) Convergence history for the exoskeleton knee joint angle in time domain, with all iterations overlapped. Here, the phase-lead compensation is γ=5, and the learning gain is φ=0.2. (**b**) Convergence plot of RMS tracking error in iteration domain. The outer learning loop is unstable for γ=0 and higher γ values (within a range) help improve the convergence.

The results of the time-domain simulation of the outer learning loop are presented in Figure 11, where the P-type learning gain was φ=0.2, and the phase-lead compensation level was γ=5.The simulated output knee angles $\theta_{O-sim,j}\left(t\right)$ of all iterations $\left(j=1,\;2,\ldots ,\;52\right)$ are overlapped with the simulated desired knee angle curve $\theta_{d-sim}\left(t\right)$ from time t=0 s to t=4.6 s in Figure 11a, which illustrates the history of convergence. It can be seen that the output knee angle $\theta_{O-sim,j}\left(t\right)$ approached the desired knee angle $\theta_{d-sim}\left(t\right)$ as the number of iteration j increased. However, there were some “unsmooth” areas of the output knee angle $\theta_{O-sim,j}\left(t\right)$, especially when the knee joint switch edits direction of rotation. Such a phenomenon is caused by the highly nonlinear nature of the Bowden transmission cables, as well as gear-reducer backlashes. In Figure 11b, the RMS (root mean square) tracking error is plotted in the iteration domain to show the convergence/divergence of error. It was found that the learning system was unstable when there was no phase-lead compensation (e.g.,γ=0   and φ=1.2, 1.4, 1.8). Additionally, higher levels of phase-lead compensation γ (within a certain range) help stabilize the learning and improve the tracking performance (e.g., γ=2, 4, 6). However, when γ is too large, the learning system becomes unstable again (e.g., γ=18).

## 5. Experimental Verification

### 5.1. Test Setup

**Figure 12:**
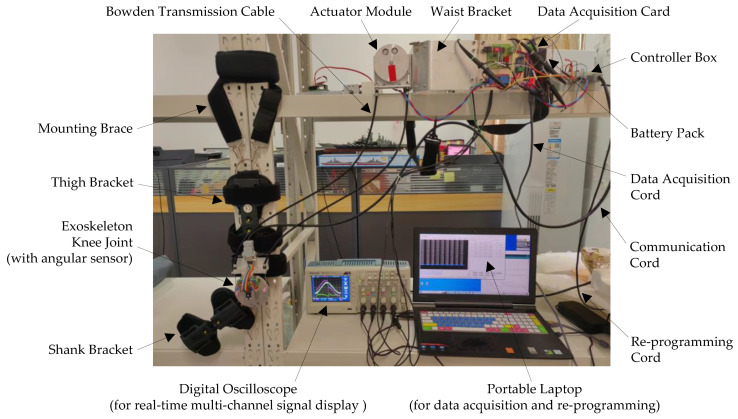
Experimental setup to test the learning-based repetitive controller for the soft knee exoskeleton with nonlinear friction and backlash of the Bowden transmission cables.

To evaluate the proposed learning-based repetitive controller for the soft knee exoskeleton with nonlinear friction and backlash of the Bowden transmission cables, an experimental setup was built. The associated mechanical structure, controller box, power source, sensors, signal displays, and acquisition modules are shown in Figure 12.

The mechanical structure consists of the shank and thigh brackets of the knee exoskeleton, the knee joint assembly, the mounting brace, the Bowden transmission cables, the actuator module, and the waist bracket. The actuator module is composed of the DC motor and the gear reducer, which is attached to the waist bracket. The model of the DC motor is XD-42GA775-24V-25W, manufactured by Xin Da Motor Corp. Ltd., with a rated output speed of 50 round × min^−1^ and built-in gear ratio of 100:1. The maximum output torque is 0.917 N × m and the weight is 540 g. The thigh bracket of knee exoskeleton was fixed to the test frame using the mounting brace, while the shank bracket was free to move. The outer sheath of the Bowden transmission cable (5 mm in outer diameter and 3 mm in inner diameter) was connected to the adjustable stops. The inner cord (2 mm in diameter) was connected to the pulleys of the knee joint and actuator module. As the actuator rotates, its motion and torque were transmitted to the knee joint of the exoskeleton, causing the shank bracket to swing around the knee axle.

**Figure 13:**
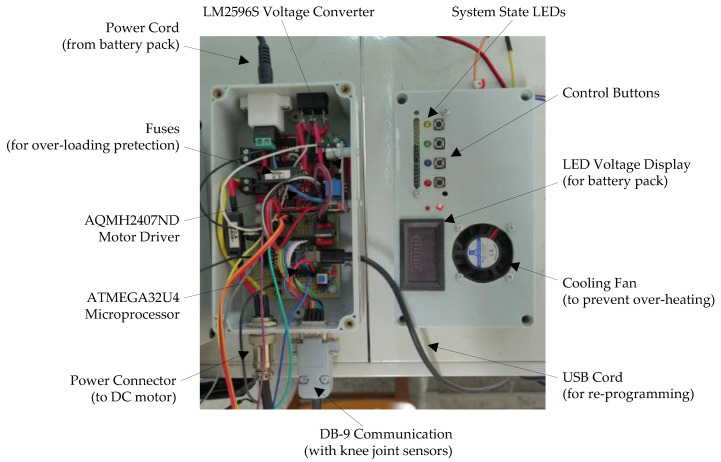
Custom-designed controller box for the soft knee exoskeleton. The ATMEGA32U4 microprocessor is used for the embedded implementation of the proposed control algorithm, and the AQMH2407ND motor amplifier is used to drive the DC motor.

The control system is a custom-built controller based on the ATMEGA32U4 microprocessor, as shown in Figure 13. The LM2596S voltage regulator was used to convert the 24 V output of the 12-cell 6000 mAH Li-ion battery pack to the 5 V input for the microcontroller and other sensors. The portable laptop compiled the C code in the Arduino IDE and programs the microprocessor through the USB re-programming cable. The output PWM signal of the microprocessor was amplified by the 160W-7A AQMH2407ND motor driver to power the DC motor through the aviation power plug. Wirings for peripherals were all integrated with the microprocessor and motor driver. A DB-9 connector was used for communications between the knee joint sensors and the microcontroller. For over-loading and over-heating protection of the motor driver, fuses were added with a cooling fan mounted on the cover of the controller box. An LED voltage display was also installed on the front panel of the controller box to monitor the supply voltage of the exoskeleton system’s battery pack in real time.

**Figure 14:**
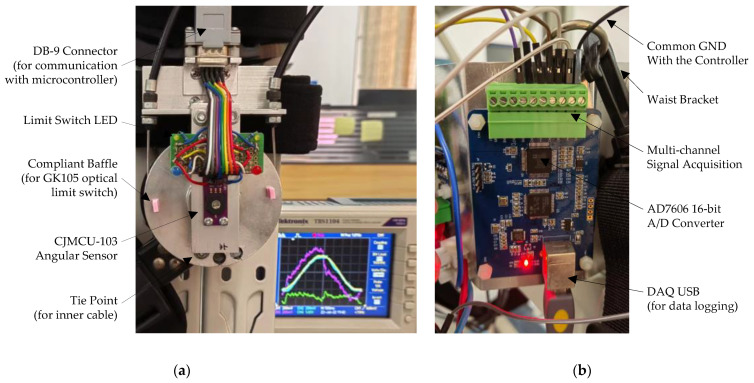
Sensors of the soft exoskeleton knee joint and the associated data-acquisition system.(**a**) Sensor system for the knee joint, with the CJMCU-103 rotary potentiometer for measuring the joint angular displacement and GK105 optical switches for limiting the swing angle of shank bracket. (**b**) Multi-channel data-acquisition system based on the 16-bit AD 7606 A/D converter, with the DAQ USB cable connected to the laptop for acquisition configuration and data logging.

The sensors and data-acquisition system for the soft exoskeleton knee joint are presented in Figure 14. As seen from Figure 14a, the CJMCU-103 rotary potentiometer was mounted on the knee joint axle and read the angular displacement of the exoskeleton knee joint. GK105 optical switches were installed on the PCB board and worked with the flexible baffles to limit the operational range of the shank bracket for user safety. The Tektronix TBS1104 oscilloscope displayed the important signals in real time, including the desired knee angle for the outer loop $\theta_{d}\left(t\right)$, the reference input knee angle $\theta_{L}\left(t\right)$ for the inner loop, the output joint angle $\theta \left(t\right)$, and the motor voltage signal $u\left(t\right)$. In Figure 14b, the multi-channel data-acquisition system is shown, where the AD7606 chip is used for the 16-bit analog-to-digital (A/D) conversion. Additionally, the DAQ USB cable communicated with the portable laptop for acquisition configuration and data logging.

To verify the proposed control algorithm in this paper, the dynamical tracking performances of the soft knee exoskeleton system using the different control methods were compared by experiments. Experimental data in Section 5.2 verify the frictional hysteresis of the knee joint angle, when only the inner-loop PD servo control was used. The test result confirms the need of the learning control method proposed in this paper. In Section 5.3, the improved tracking performance of the proposed learning-based repetitive controller is verified by additional tests. Additionally, the learning controller with different levels of phase-lead compensation is further compared and analyzed, in terms of both the tracking performance of knee joint angle and the smoothness of the motor signal.

Before running the test, tensions of the inner cable should be adjusted properly. Instead of creating ultra-high pretensions, the inner cables were pre-tightened just to remove the major slackness (with small amount of backlash left). Such a procedure mimics the real-world application of the soft knee exoskeleton, where the Bowden cables might become loose over time due to repeated usage. Moreover, lower cable pretensions help reduce the unnecessary frictional loss of the entire Bowden cable transmission system.

### 5.2. PD Control of the Inner Loopand Verification of Frictional Hysteresis

To illustrate the frictional hysteresis of the Bowden transmission cable with only the inner-loop PD controller, a sinusoidal signal is used in this article as the reference input knee angle $\theta_{L}\left(t\right)$, which is formulated as

(51) $$\theta_{L}\left(t\right)=A\sin\left(2\pi ft\right)+D$$

where the frequency is f=0.2174 Hz (period of 4.6 s), the amplitude is A=28.8 deg (57.6 deg pk-pk), and the bias term is D=45 deg.

**Figure 15:**
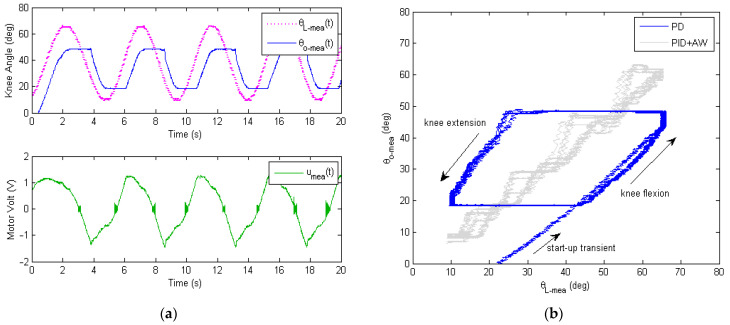
Measured tracking performance of the inner-loop digital servo control of the soft knee exoskeleton. (**a**) Experimental data of the sinusoidal response of the soft knee exoskeleton, with the motor control signal shown. (**b**) Experimental data of the reference input knee angle $\theta_{L-mea}\left(t\right)$ v.s. the output knee angle $\theta_{O-mea}\left(t\right)$.

The experimental results of the soft exoskeleton’s tracking performance are shown in Figure 15, which are very similar to the simulation results in Figure 7. In the upper plot of Figure 15a, the measured output knee angle $\theta_{O-mea}\left(t\right)$ (in blue) lags behind the sinusoidal reference input knee angle $\theta_{L-mea}\left(t\right)$ (in magenta). Furthermore, a significant amount of distortion existed in the output knee angle, where the top and bottom part were truncated due to Bowden cable friction. The lower plot of Figure 15a shows the time history of the measured motor control signal. Since the inner-loop PD controller generates the motor control signal $u\left(t\right)$ only based on the servo error $e_{S}\left(t\right)$ of current and previous time steps, the DC motor was unable to compensate for the frictional disturbances of the Bowden cables. Figure 15b further shows in the X–Y plot the strong hysteresis and backlash caused by the friction in the Bowden transmission cables. Here, the tracking results using PID + AW are also presented (in gray). It can be seen that the integral action with anti-windage helps improved the tracking performance to some extent, as predicted by the simulation in Figure 7b. However, due to the nonlinear frictional hysteresis and backlash, the choppy response of the PID + AW control makes it undesirable to the smooth and safe operation of the soft exoskeleton.

### 5.3. Phase-Lead Iterative Learning Control of the Outer Loop

#### 5.3.1. Overall History of the Learning Process

**Figure 16:**
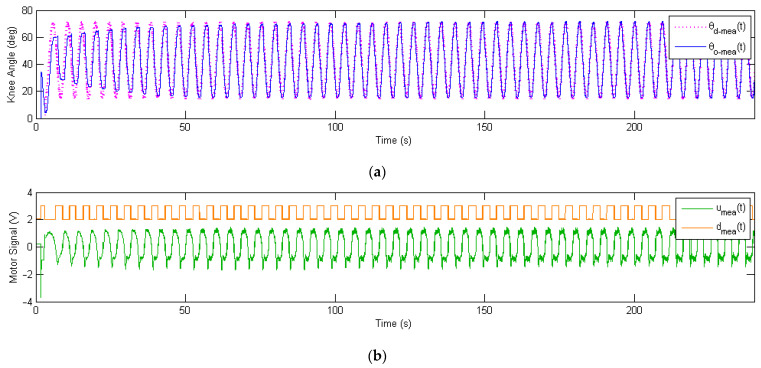
Experimental tracking performance of the outer learning loop for the soft knee exoskeleton, showing a total of 52 repetitions in the time domain. The P-type learning gain is φ=0.25, and the phase-lead compensation level is γ=10. (**a**) Measured sinusoidal response of the soft knee exoskeleton, with the desired knee angle $\theta_{d-mea}\left(t\right)$ in magenta and output knee angle $\theta_{O-mea}\left(t\right)$ in blue. (**b**) Measured motor control signal, showing both the motor voltage signal $u_{mea}\left(t\right)$ in green and the motor direction signal $d_{mea}\left(t\right)$ in orange.

To address the hysteresis issue of the inner PD control loop above, a phase-lead iterative learning control (PLILC) of the outer loop wasproposed and validated by experiments. The time history of the entire 52 iterations is presented in Figure 16, where theP-type iterative learning gain is φ=0.25 and the phase-leadlevel is γ=10.

The measured output knee angle $\theta_{O-mea}\left(t\right)$ wasplotted (in blue) and compared with the desired knee angle $\theta_{d-mea}\left(t\right)$ (in magenta), as shown in Figure 16a. For the first few iterations, the phase of measured output knee angle $\theta_{O-mea}\left(t\right)$ lagged behind the desired knee angle $\theta_{d-mea}\left(t\right)$, where the amplitude was lower and the profile was distorted due to Bowden cable friction. As the soft exoskeleton repeated the sinusoidal track following for more repetitions, the output knee angle $\theta_{O-mea}\left(t\right)$ approached the desired knee angle $\theta_{d-mea}\left(t\right)$ more and more closely. Figure 16b presents the time history of the measured motor control signal $u_{mea}\left(t\right)$ (in green) and motor direction signal $d_{mea}\left(t\right)$ (in orange). Instead of staying roughly unchanged as in the PD control loop (Figure 15a), the motor signals $u_{mea}\left(t\right)$ and $d_{mea}\left(t\right)$ updated themselves as the number of repetitions increased, with the help of learning. The update of the motor signal led to the accurate tracking of the desired knee angle $\theta_{d-mea}\left(t\right)$ as shown in Figure 16a.

#### 5.3.2. Analysis of the Output Response

**Figure 17:**
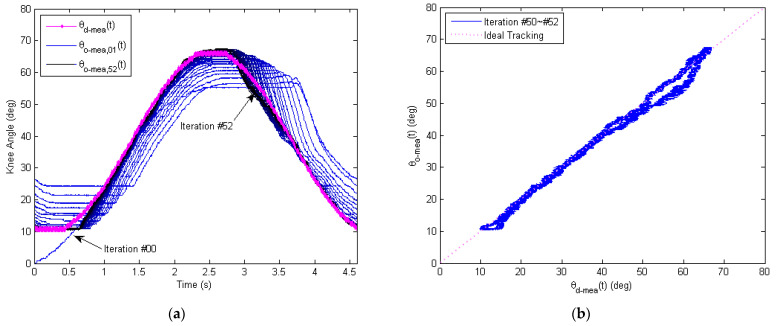
Measured tracking performance using the proposed learning-based repetitive controller, where the learning gain is φ=0.25, and the phase-lead compensation level is γ=10. (**a**) Convergence history for the exoskeleton knee joint angle in time domain, with iterations #00 to #52 overlapped. (**b**) X–Y plot of the desired knee angle $\theta_{d-mea}\left(t\right)$ against the measured output knee angle $\theta_{O-mea}\left(t\right)$ for iterations #50 to #52. With the outer learning loop, the frictional hysteresis of the Bowden transmission cable is significantly reduced after learning, as compared with Figure 15b where only the inner PD loop is active.

A detailed analysis of the experimental tracking performance is presented Figure 17. The convergence history of the measured output knee angle $\theta_{O-mea,j}\left(t\right)$ is depicted in Figure 17a, where all iterations (j=#00 to #52) are plotted together in one period (for t = 0 s to 4.6 s) and compared with the desired knee angle $\theta_{d-mea}\left(t\right)$ in magenta. To demonstrate the process of convergence, the plot color of the measured output knee angle $\theta_{O-mea,j}\left(t\right)$ evolves from light blue (for the initial iterations) to dark blue (for the final iterations). As can be seen from Figure 17a, the phase lag and amplitude distortion caused by the Bowden cable friction and gear-reducer backlash are both decreased as more and more iterations were learnt.

The tracking performance for the final iterations (#50 to #52) is further illustrated as an X–Y plot in Figure 17b, where the desired knee angle $\theta_{d-mea}\left(t\right)$ is plotted along the *X*-axis and the measured output knee angle $\theta_{O-mea,j}\left(t\right)$ is plotted along the *Y*-axis. For the ideal and perfect tracking, the X–Y plot is a straight line with a slope of 45 deg from the origin (dotted line in magenta). As seen from Figure 17b, the actual measured X–Y plot stayed mostly close to the ideal line for the final iterations. However, the RMS tracking performance of the knee flexion cycle (~0.916 deg) was a little better than that of the knee extension cycle (~1.734 deg).In particular, at the beginning of the knee extension cycle, the output knee angle $\theta_{O-mea,j}\left(t\right)$ lagged behind the desired knee angle $\theta_{d-mea,j}\left(t\right)$ at first and then quickly moved ahead until it tracked closely again. One possible cause is the gravitation disturbance, which over-assists the motion of knee extension when the motor torque surpasses the maximum static friction in the Bowden transmission cable. Another factor leading to this result is the bending angle difference of the flexion and extension cables that produce different Bowden cable friction for the flexion and extension cycles. Furthermore, asymmetric frictional and viscous resistances inside the gear reducer during the clockwise and counter-clockwise rotations might also contribute to this phenomenon. Despite these observed imperfections, the overall frictional hysteresis of the Bowden transmission cable was significantly reduced after learning, as compared with Figure 15b with only the inner-loopPD control.

#### 5.3.3. Mechanism of the Learning Compensation

**Figure 18:**
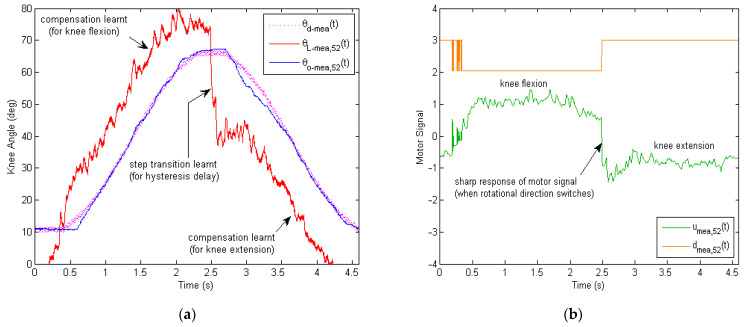
Analysis of the measured learning compensation for the inner loop. Sharp-step transitions weregenerated through learning over repetitions, which compensate for the hysteresis delay caused by the Bowden cable friction and gear-reducer backlash. (**a**) Comparison of the learnt reference input knee angle $\theta_{L-mea}\left(t\right)$ for the inner PD control loop and the desired knee angle $\theta_{d-mea}\left(t\right)$ for the outer loop, at iteration #52. (**b**) Time history of the motor control signal $u_{mea}\left(t\right)$ and motor direction signal $d_{mea}\left(t\right)$, at iteration #52.

The essence of learning is the iterative update of the reference input knee angle signal $\theta_{L-mea}\left(t\right)$ for the inner PD control loop. When there was no learning, the reference input signal $\theta_{L-mea}\left(t\right)$ maintained the same as the desired knee angle command $\theta_{d-mea}\left(t\right)$ for all repetitions. When learning was implemented, the reference input knee angle signal $\theta_{L-mea}\left(t\right)$ changed from repetition to repetition, based on the tracking error $e_{L-mea}\left(t\right)$ and reference input knee angle $\theta_{L-mea}\left(t\right)$ of the previous repetition j−1.The design of the learning-based repetitive controller lends itself toward the the optimal tracking of the desired knee angle $\theta_{d-mea}\left(t\right)$, including the speed of convergence, the learning transients, and the final error levels. Moreover, the complexity of the learning control algorithm and the computational time involved are of equal importance for practical implementations in real time. In this article, a basic, yet effective P-type iterative learning law with a phase-lead compensator was proposed to address the issue of frictional hysteresis of the Bowden transmission cables. The computation time of the proposed learning algorithm took around 0.010 s, which allows for 0.015 s of the 12-bit A/D sampling and other activities of the ATMEGA32U4 microprocessor. The detailed learning mechanism is illustrated in Figure 18.

The updated reference input knee angle signal $\theta_{L-mea,j}\left(t\right)$ at iteration j=52 is plotted in red, as Figure 18a shows, where the learning gain φ=0.25 and the phase-lead level γ=10. The phase-lead compensator is of particular importance for the stability of learning and final tracking performance. It helps compensate for the nonlinear hysteresis delay and allows the outer loop to learn at higher frequencies fora more aggressive learning profile of $\theta_{L-mea,52}\left(t\right)$. It can be seen that $\theta_{L-mea,52}\left(t\right)$ was quite different from the desired knee angle signal $\theta_{d-mea}\left(t\right)$ at iteration #52. The learnt reference knee angle $\theta_{L-mea,52}\left(t\right)$ was greater than the desired knee angle $\theta_{d-mea}\left(t\right)$ during the knee flexion cycle, and $\theta_{L-mea,52}\left(t\right)$ was smaller than $\theta_{d-mea}\left(t\right)$ during the knee extension cycle. The discrepancy between $\theta_{L-mea,52}\left(t\right)$ and $\theta_{d-mea}\left(t\right)$ led to the learnt compensation for the inner PD control loop, which helped correct the output knee angle $\theta_{O-mea,52}\left(t\right)$ of the soft exoskeleton for accurate tracking. Here, the step transition of the learnt reference knee angle $\theta_{L-mea,52}\left(t\right)$ is very crucial for dealing with the hysteresis delay caused by the Bowden cable friction and gear-reducer backlash.

With the step transition in the reference knee angle signal $\theta_{L-mea,52}\left(t\right)$, the associated motor control signal $u_{mea,52}\left(t\right)$ generateda sharp response when its direction of rotation $d_{mea,52}\left(t\right)$ switched from knee flexion to knee extension, as illustrated in Figure 18b.Unlike the inner-loop PD control alone, where the motor signal changes gently (Figure 15a), the sharp response of motor control signal (after learning) raises/lowers the motor voltage very quickly when needed. As a result, the Bowden cable friction could be immediately surpassed by the output motor torque to minimize any unnecessary hysteresis delays.

#### 5.3.4. Evaluation of the Tracking Error

**Figure 19:**
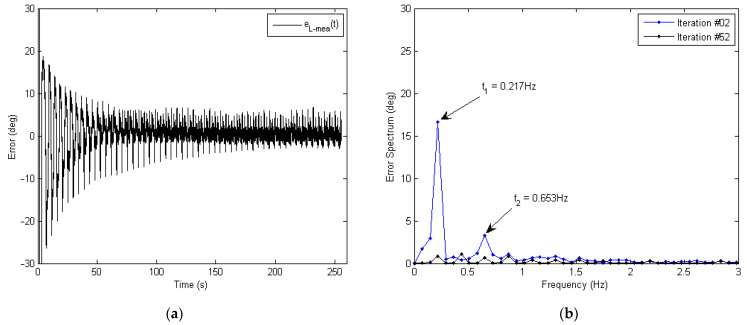
Convergence of the measured tracking error for the outer learning loop, where the learning gain is φ=0.2, and the phase-lead compensation level is γ=10. (**a**) Time-domain convergence of the measured knee joint tracking error $e_{L-mea}\left(t\right)$. (**b**) Frequency-domain comparison of the tracking error spectrum $E_{L-mea}\left(f\right)$ at iterations #02 and #52. After learning, the dominant error components at frequencies $f_{1}=0.217\;\mathrm{Hz}$ and $f_{2}=0.653\;\mathrm{Hz}$ wereattenuated by 95.1% and 80.2%, respectively.

To quantitatively evaluate the tracking performance of the proposed learning controller, the tracking error $e_{L-mea}\left(t\right)$ was obtained based on the difference between the desired knee angle $\theta_{d-mea}\left(t\right)$ and the measured output knee angle $\theta_{O-mea}\left(t\right)$, as shown in Figure 19.

The overall history of the tracking error $e_{L-mea}\left(t\right)$ in the time domain is plotted in Figure 19a. For initial iterations, the tracking error was relatively large, which had an amplitude of approximately 20 deg (pk-pk). When more iterations were learnt, the tracking error started decreasing, showing the stable convergence of learning. Additionally, for the final iterations, the tracking error roughly stayed within a range and mostly consisted of noise.

Figure 19b further analyzes the tracking error $e_{L-mea}\left(t\right)$ in the frequency domain. Two dominant peaks at frequencies $f_{1}=0.217\;\mathrm{Hz}$ and $f_{2}=0.653\;\mathrm{Hz}$ were found in the error spectrum $E_{L-mea,2}\left(f\right)$ (in light blue) for the initial iteration (#02). After learning, these two dominant frequency components were significantly attenuated, as seen from the spectrum $E_{L-mea,52}\left(f\right)$ (in dark blue) for the final iteration (#52). The percentages of reduction for these two dominant frequency components were 95.1% and 80.2%, respectively. The result of the error spectrum verifies the ability of the outer learning loop for reducing the tracking errors, particularly the low-frequency error components. As the bio-mechanical motion frequency of a human’s lower limbs are mostly in the low-frequency range (below 6.21 Hz [49,50]), the high-frequency error components are normally of less importance for the operation of a soft exoskeleton.

#### 5.3.5. Comparison of Different Control Methods

**Figure 20:**
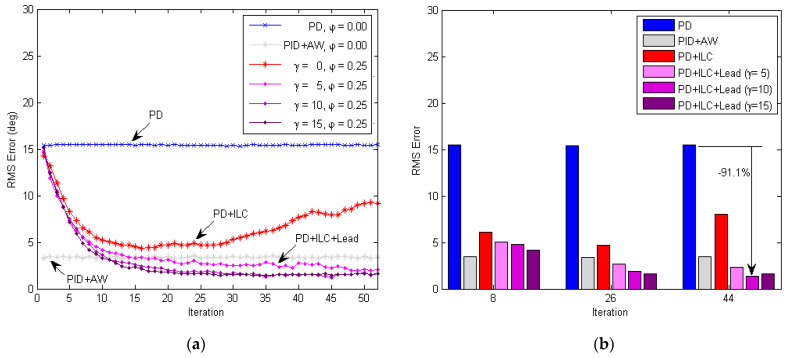
Comparison of the RMS tracking error using different control methods. (**a**) Iteration-domain convergence of the RMS knee joint tracking error. The RMS error of the PD and PID + AW control stays roughly unchanged over repetitions as expected. The RMS error of PD + ILC control decreases for the first 17 iterations and then diverges. The RMS error of PD + ILC + Lead control converges monotonically at different speeds. (**b**) Comparison of the RMS tracking error at different iteration numbers (#8, #26 and #44). Overall, the PD + ILC + Lead (γ=10) performs the best, achieving 91.1% error reduction at iteration #44, compared with the PD control.

The tracking performances of different control approaches were compared using the RMS tracking error, as shown in Figure 20. The process of convergence for the RMS error in the iteration domain (i.e., with respect to the number of iterations) is presented in Figure 20a. It can be seen that the RMS error of the inner-loop PD controller alone maintained aconstant level around 15.5 deg. The RMS error using the PID+AW controller was reduced significantly to around 3.4 deg. However, the response of the angular displacement was rather choppy using PID+AW control as seen from Figure 15b, which is undesirable for the smooth and safe operation of the soft exoskeleton. Since there is no learning for the either the PD or PID+AW controller itself, the tracking performance did not improve as the number of repetitions increased. The RMS error of the PD+ILC method (φ=0.25) decreased initially to 4.31 deg at iteration #16 but then diverged. Smaller learning gains were also tested and similar results/trends were obtained for the PD+ILC approach. As a result, the PD+ILC method is unstable and ineffective in controlling the soft exoskeleton due to the large phase delay caused by the friction of the Bowden transmission cables.

In response to this issue, a phase-lead compensator with level γ is introduced. With the lead compensation, the learning-based repetitive control could adjust the reference input knee angle $\theta_{L-mea}\left(t\right)$ a few time steps ahead for the Bowden cable transmission system to catch up with the change of the desired knee angle signal $\theta_{d-mea}\left(t\right)$. As Figure 20a shows, the control method using PD + ILC + Lead was stable for γ=5, 10, 15. Here, the RMS tracking error was relatively higher for γ=5 and almost similar for γ=10 and 15. The speed of convergence increased with the value of γ, which is consistent with the simulation results in Figure 11b.

Figure 20b further compares these different control methods at three different iteration numbers to evaluate the transient performance of learning. At iteration #08, the RMS tracking errors of PD + ILC + Lead control (with γ=0, 5, 10, 15) all converged, where the lowest was4.21 deg for γ=15. At iteration #26, the RMS errors further decreased, and the learning control with γ=15 still produced the lowest RMS error of 1.58 deg. At iteration #44, the RMS error of PD + ILC control (i.e.,γ=0) diverged to 8.02 deg, and the learning with γ=10 waslowest in RMS error at 1.37 deg. Higher γ values were also tested, but the motor signal became noisier and less stable as predicted by the simulation results in Figure 11b, which is not beneficial to the safe and smooth operation of the exoskeleton. As a whole, the PD + ILC + Lead with γ=10 offers the best performance in terms of speed of convergence and the final RMS tracking error level, as summarized in Table 2 below.

## 6. Conclusions

This article proposed a learning-based repetitive controller which could compensate for the nonlinear cable friction and gearbox backlash in an iterative manner. The presented control method does not rely on the accurate modeling of the friction and backlash characteristics of the Bowden transmission system as required by many of the existing methods. Instead, it applies iterative learning to adaptively update the reference trajectory based on the internal model principle (IMP), which minimizes the output hysteresis caused by cable friction and gearbox backlash. The direct application of the ILC to the PD feedback control loop, however, is unstable due to the considerable phase delay caused by friction and backlash. As a result, a digital phase-lead compensator was designed and integrated with the ILC for improved stability and tracking accuracy. With the proposed phase-lead iterative learning control (PLILC) design, the tracking performance of knee angle was greatly improved, where a reduction of 91.1% in RMS error was achieved, as compared with the conventional PD control. While the PID controller with anti-windage could also improve the tracking performance, the choppy response and intensive parameter tuning required makes it undesirable for the safe and smooth operation of soft exoskeletons. The proposed method is less sensitive to model uncertainties, and the RMS tracking error (~1.37 deg) is very close to other control methods which rely on the complex modeling of the Bowden cable system. Furthermore, the result is a significant advancement considering the limited power output of the actuator and finite quantization resolution of the microprocessor being used in this study. One limitation of this article is that the disturbance of human body is not considered in particular during the test, which will be considered in future research. Collaborations with the General Hospital of the Eastern Theater Command are also underway for the rehabilitation of orthopedic trauma, and additional work is needed for the proposed equipment to conform to the clinical requirements. Not with standing the limitation, the proposed learning-based repetitive control design offers promising options for the realization of lightweight and low-cost wearable exoskeletons with high tracking accuracies.

## Figures and Tables

**Table 1:** Weights and dimensions of the major exoskeleton components.

Exoskeleton Component	Weight (g)	Size ^1^ (mm)
Waist Bracket	260	1000 (C) × 100 (W) × 1 (H)
Thigh Bracket	321	300 ± 50 (L) × 75 (W) × 15 (H)
Shank Bracket	254	250 ± 50 (L) × 75 (W) × 15 (H)
Back Strap	12 × 2	800 ± 20 (L) × 25 (W) × 2 (H)
Actuator Module	778	120 (L) × 80 (W) × 80 (H)
Battery Pack	782	150 (L) × 90 (W) × 40 (H)
Controller Box	316	150 (L) × 90 (W) × 60 (H)
Bowden Cable (Outer Sheath)	20 × 2	600 (L) × 5 (D)
Bowden Cable (Inner Cord)	8 × 2	1000 (L) × 2 (D)
Grooved Pulley	136 × 2	80 (D) × 10 (H)

^1^ L: length; W: width; H: height; D: diameter.

**Table 2:** Comparison of the RMS tracking error (unit: deg)using different control methods.

Control Method	RMS Error (Iteration #8)	RMS Error (Iteration #26)	RMS Error (Iteration #44)	Iterations for Convergence	Minimum RMS Error	Stability
PD	15.512	15.351	15.454	n/a	15.312	Yes
PID + AW	3.478	3.411	3.499	n/a	3.235	Yes
PD + ILC	6.081	4.665	8.029	n/a	4.314	No
PD + ILC + Lead (γ= 5)	5.049	2.632	2.291	8	1.957	Yes
PD + ILC + Lead (γ=10)	4.739	1.881	1.371	7	1.208	Yes
PD + ILC + Lead (γ=15)	4.213	1.579	1.601	6	1.456	Yes

## Data Availability

The data used to support the findings of this study are available from the corresponding author upon request.
